# Variation of Pro- and Anti-Inflammatory Factors in Severe Burns: A Systematic Review

**DOI:** 10.3390/ijms262010131

**Published:** 2025-10-17

**Authors:** Mihai-Codrin Constantinescu, Mihaela Pertea, Stefana Avadanei-Luca, Alexandru-Hristo Amarandei, Andra-Irina Bulgaru-Iliescu, Malek Benamor, Dan Cristian Moraru, Viorel Scripcariu

**Affiliations:** 1 Faculty of Medicine, Grigore T. Popa University of Medicine and Pharmacy Iasi, 700115 Iași, Romania; 2 Department of Plastic Surgery and Reconstructive Microsurgery, “Sf. Spiridon” Emergency County Hospital, 700111 Iași, Romania; alexamarandei@yahoo.com (A.-H.A.); benamor120@gmail.com (M.B.); 3 First Surgical Oncological Unit, Regional Institute of Oncology, 700483 Iași, Romania

**Keywords:** pro-inflammatory mediators, anti-inflammatory mediators, cytokines, burn injury, systemic inflammation, biomarkers, prognosis

## Abstract

Burn injury triggers a complex inflammatory cascade in which the interplay between pro- and anti-inflammatory mediators determines recovery or progression to sepsis, ventilator-associated pneumonia (VAP) or multi-organ dysfunction, and mortality. We systematically searched PubMed, Embase, Cochrane Library, Web of Science, and Scopus for studies published between 2006 and 2024, identifying 1883 records. We conducted a comprehensive systematic review in accordance with the Preferred Reporting Items for Systematic Reviews and Meta-Analyses (PRISMA) guidelines. After screening and eligibility assessment, 24 studies covering both pediatric and adult populations met the inclusion criteria. Data on cytokines, acute-phase proteins, complement fragments, and systemic inflammatory indices were synthesized narratively. The evidence indicates that the inflammatory response to burn injury is not a linear sequence of events but a dynamic and unstable equilibrium, where outcomes are determined less by the initial magnitude of cytokine release and more by the persistence of dysregulated inflammation or failure of compensatory mechanisms.

## 1. Introduction

Burn wound healing occurs in four overlapping stages. First, hemostasis, mediated by platelet activation, prevents further blood loss. This is followed by a strong local inflammatory response, which recruits immune cells to the wound. The third stage, proliferation, is characterized by keratinocyte migration, fibroblast activity, and angiogenesis. Finally, remodeling involves extracellular matrix reorganization and macrophage polarization toward the reparative M2 phenotype [1]. Neutrophils are the first cells attracted to the site of injury under the action of cytokines and chemokines released by platelets in the first hours after the burn occurs [1]. In addition to their role in phagocytosing microbes, necrotic cells, and debris from the extracellular matrix, neutrophils also release tumor necrosis factor α (TNF-α), interleukin-1 beta (IL-1β), and IL-6, which attract monocytes and macrophages [1]. The migrated and activated cells release chemotactic mediators, growth factors, and signaling molecules that stimulate keratinocyte and fibroblast activity during the proliferation phase. In the subsequent remodeling phase, macrophages differentiate toward the M2 phenotype, promoting tissue repair and extracellular matrix restoration [1].

Local inflammation is essential for healing injuries and prevents the development of pathogens. However, this process must be well regulated, as chronic inflammation in severe injuries significantly delays tissue repair or leads to pathological scarring [2]. From the first day after the burn, patients showed significant increases in interferon γ (IFN-γ), IL-1β, IL-1Ra, IL-2, IL-4, IL-7, IL-9, IL-10, IL-12p70, IL-13, IL-17, macrophage inflammatory protein (MIP-1α), MIP-1β, platelet-derived growth factor subunit B (PDGF-BB), vascular endothelial growth factor (VEGF), basic fibroblast growth factor (FGF), and Eotaxin; however, the values measured at day 3 had no predictive value for survival [3]. Interleukins IL-6, IL-8, and MCP-1 increase rapidly in the first 4 days and persist for several weeks, triggering the release of acute phase proteins from the liver, influencing T-lymphocytes function, and angiogenesis. CRP also increases sharply, persisting for several months after the burn. IL-1Ra and IL-10, anti-inflammatory cytokines, increase from the first day but gradually decrease over several weeks [1,4,5].

In severe burns, the body reacts with a massive inflammatory response, releasing huge amounts of pro-inflammatory cytokines (IL-6, IL-8, IL-1β, monocyte chemotactic protein-1 (MCP-1), MIP-1β, IL-13, IL-2, IL-7, and Granulocyte-Macrophage Colony-Stimulating Factor, GM-CSF), whose effect will subsequently be counteracted by the production of anti-inflammatory cytokines IL-10, Granulocyte Colony-Stimulating Factor (G-CSF), IL-17, IFN-γ, IL-4, and IL-12 p70. Cytokines are indispensable for tissue repair and protection against infections, as they are involved in the recruitment and activation of immune cells, as well as angiogenesis, cell proliferation, and apoptosis [6]. Early increases in MCP-1, IL-6, IL-8, and IL-10 were significantly correlated with 28-day mortality in burn patients [7]. In parallel, a recent meta-analysis confirmed that elevated admission Neutrophil-to-Lymphocyte Ratio (NLR) is an independent predictor of mortality in burn patients [8]. Beyond cytokines and acute-phase proteins, complement activation has been shown to contribute to burn-induced inflammation, while neutrophil extracellular trap (NET) formation has been implicated in tissue injury and immune dysregulation following burns. These mechanisms are increasingly recognized as important enhancers of the systemic inflammatory response [9,10,11].

This review summarizes studies that analyze quantitative measurements of circulating cytokines, acute-phase reactants, complement activation fragments, or hematological indices, and correlate them with clinical outcomes such as sepsis, ventilator-associated pneumonia (VAP), multi-organ dysfunction, or mortality.

Nonetheless, significant knowledge gaps persist regarding which aspects of the burn-triggered inflammatory response are most amenable to therapeutic intervention. Restoring the balance between pro- and anti-inflammatory forces is key to improving outcomes in severe burns, yet no targeted immunomodulatory therapy has been established for burn patients. Notably, therapies targeting specific cytokines have proven beneficial in other hyperinflammatory conditions (for example, IL-6 antagonism in severe COVID-19), suggesting that similar approaches could be explored in burns. Therefore, to address these gaps, we undertook this review to identify which pro- and anti-inflammatory mediators are consistently associated with adverse outcomes in severe burns and thereby inform potential targets for intervention.

## 2. Materials and Methods

### 2.1. Protocol and Reporting Framework

This systematic review was conducted in accordance with the Preferred Reporting Items for Systematic Reviews and Meta-Analyses (PRISMA) 2020 guidelines for systematic reviews [12]. The review protocol was prospectively registered in the International Prospective Register of Systematic Reviews (PROSPERO; ID CRD420251162183). In addition, given the breadth and heterogeneity of outcomes and measurement methods across the included observational studies, this work is a systematic review with narrative synthesis conducted under PRISMA-2020.

While not a scoping review, the narrative mapping of evidence shares reporting overlap with PRISMA-ScR items; we therefore ensured clarity on scope and eligibility throughout and followed a completed PRISMA 2020 checklist to guide reporting (Table S1).

### 2.2. Research Question and Eligibility Criteria

The primary objective of this review was to examine the temporal patterns of circulating pro- and anti-inflammatory mediators and hematologic indices in burn patients, and to evaluate their associations with major clinical outcomes, including sepsis, ventilator-associated pneumonia, organ dysfunction, and mortality. We included human studies (adults or children) with severe burn trauma that reported quantitative measurements of circulating cytokines, acute-phase reactants, complement activation fragments, or hematologic indices and correlated them with clinical outcomes. Exclusion criteria included studies without an available full text in English, those based on animal or in vitro experiments, reviews, case reports, commentaries, and studies that did not report at least one pro- or anti-inflammatory factor.

The definition of “severe burns” varied among the included studies, with total body surface area (TBSA) thresholds typically ranging from >20% to >40%. To ensure inclusivity and comparability, we accepted each study’s definition of severity as reported by the authors, provided that the population represented patients with clinically significant burn injury requiring specialized or intensive care.

### 2.3. Information Sources and Search Strategy

The systematic literature search was conducted in PubMed, Embase, Cochrane Library, Web of Science, and Scopus, covering publications between January 2006 and March 2024. The strategy combined keywords and MeSH terms related to “burn injury”, “cytokines”, “inflammatory mediators”, and “systemic inflammation”.

### 2.4. Study Selection Process

**Figure 1:**
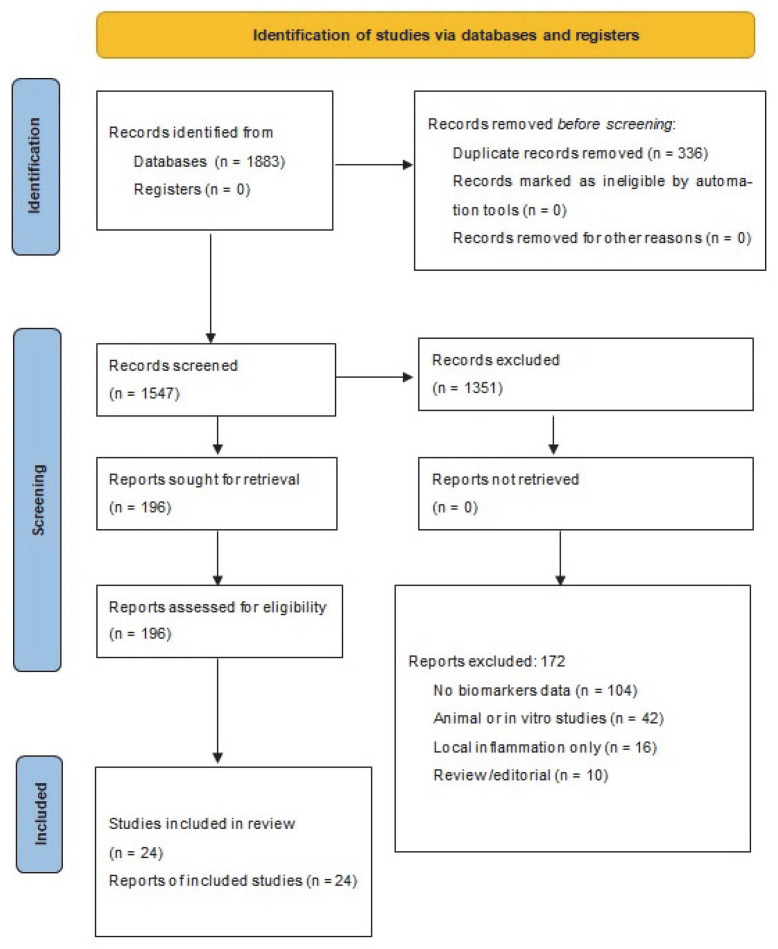
PRISMA 2020 flow diagram.

The literature search identified a total of 1883 records. After removal of duplicates, 1547 unique studies remained for title and abstract screening. Two reviewers independently screened all titles and abstracts retrieved by the search. Of these, 1351 were excluded as irrelevant to the inclusion criteria. The remaining 196 full-text articles were assessed for eligibility using the predefined inclusion and exclusion criteria. Disagreements were resolved by consensus. Reasons for exclusion at the full-text stage were recorded and are summarized in Figure 1. Of the 172 full-text articles excluded, 104 lacked biomarker data, 42 involved animal or in vitro models, 16 focused on local wound inflammation without systemic outcomes, and 10 were reviews or editorials. No automation tools were used in the screening process; therefore, this category in the PRISMA flow diagram is not applicable (n = 0). The higher number of animal or in vitro studies excluded reflects a stricter reclassification applied during the revised screening process, in accordance with the refined eligibility criteria.

### 2.5. Data Extraction and Synthesis

Data were extracted using a standardized form that included study design, population characteristics, timing of sampling, biomarkers assessed, and reported associations with outcomes. Owing to substantial differences in study design, patient populations, timing of sampling, laboratory techniques, and outcome definitions, a formal meta-analysis was not feasible. Nevertheless, to systematically explore heterogeneity, a structured narrative synthesis was conducted. Subgroup patterns were qualitatively examined across age categories (pediatric vs. adult), burn severity (moderate vs. extensive), and outcome domains (mortality, sepsis, ventilator-associated pneumonia, organ dysfunction). Consistency of findings across independent cohorts and analytical approaches was evaluated as a conceptual form of sensitivity analysis to assess robustness. Results were synthesized narratively and organized into the following domains: pro-inflammatory cytokines, anti-inflammatory cytokines, and cellular ratios derived from hematology and systemic inflammation indices.

### 2.6. Risk of Bias and Quality Appraisal

Due to the heterogeneity of study designs and methodologies, a structured narrative appraisal of study quality was performed, focusing on study design, sample size, assay methodology, and adjustment for confounders. In addition, a formal, domain-based risk-of-bias assessment was conducted using the Newcastle-Ottawa Scale (NOS) for observational studies, which evaluates selection, comparability, and outcome domains (maximum 9 points in total). Two reviewers independently evaluated each included study, and disagreements were resolved by discussion. Each study was assigned an overall NOS quality category: high (≥7), moderate (4–6), or low (<4). Among the 24 included studies, 10 were rated as high quality, 9 as moderate, and 5 as low. These qualitative judgements were used to contextualize the narrative synthesis and interpret the strength of the available evidence. Although detailed numerical scores are not displayed, the NOS framework provided a structured, transparent, and reproducible evaluation of methodological quality across the included studies.

## 3. Results

The literature search identified 1883 records. After removing duplicates, 1547 unique studies were screened by title and abstract, and 196 full-text articles were assessed for eligibility. Following application of the inclusion and exclusion criteria, 24 studies met all requirements and were retained for detailed analysis. The study selection process is illustrated in Prisma diagram (Figure 1).

We evaluated the cytokine dynamics, including study populations and main findings (Table 1). The assessment of hematological ratios (such as NLR, CRP, and PCT) and acute phase reactants was reviewed in Table 2. Table 3 highlights studies addressing immune cell function and related mediators.

Together, these tables provide a complementary overview of the systemic inflammatory response after severe burns, with outcomes including associations with sepsis, ventilator-associated pneumonia (VAP), multi-organ dysfunction and mortality.

### 3.1. Pro-Inflammatory Cytokines

Burn surface itself is a key determinant: even moderate burns (20–40% TBSA) triggered systemic IL-6, IL-8, TNF-α, and MCP-1 responses nearly comparable to those observed in severe burns involving >40% TBSA [32]. Classically, high IL-6 levels have been associated with poor outcomes in burn cohorts [23]. IL-6 increased rapidly within 24–48 h after burn and was significantly higher in non-survivors from day 0–1 onward [14,19]. Persistent elevation during the first week was linked with sepsis and mortality [19]. After burn injury, MCP-1 and IL-6 were markedly elevated in adipose tissue, both in mechanistic animal models [33] and in human skin biopsies [34]. These increases were associated with macrophage infiltration, linking local adipose inflammation to systemic hyperinflammation and metabolic dysfunction such as insulin resistance.

In mechanically ventilated adults with burn injury, IL-8 (but not IL-6 after adjustment) independently predicted ventilator-associated pneumonia (VAP) and mortality, with levels measured on days 0, 3, and 7 remaining significant predictors of poor outcomes [21]. IL-6 concentrations correlated with burn size (%TBSA) [6,14], whereas IL-8 persisted longer and was independently associated with pulmonary complications and mortality in ventilated patients [21]. In pediatric cohorts, IL-6, IL-8, MCP-1, and G-CSF were markedly elevated in non-survivors (up to 10,000-fold), and IL-8 levels differentiated survivors from non-survivors after days 8–10 [14,16,19]. Together, these findings highlight complementary roles of IL-6 and IL-8 in shaping the early systemic inflammatory response after severe burns [6,14,16,19,21].

Regarding systemic and local IL-1β increases, levels rose early after burn, with peaks occurring at admission in some adult cohorts [7] and later during the first week in pediatric patients [6]. Release of IL-1β is tightly linked to NLRP3 inflammasome activation; persistent activation of this pathway fuels sterile inflammation and multi-organ dysfunction [35].

Very early MCP-1 (CCL2) elevation was noted, significantly higher in non-survivors from day 1 and persisting through day 5 [14]. In children, MCP-1 rose dramatically within 24–48 h and correlated with burn size [16]. MCP-1 increased early in circulation [14,16]; in mechanistic animal models it promoted macrophage infiltration into adipose depots [33]. In severely burned children, MIP-1β (CCL4) was elevated in non-survivors at multiple time points, although it was less discriminatory than IL-6, IL-8, or MCP-1 [16]. In mechanistic models, MCP-1 and IL-8 promoted macrophage and neutrophil infiltration into adipose depots, linking local adipose inflammation with systemic hypermetabolism [33]. GM-CSF showed a transient early rise after burn, being significantly elevated in non-survivor children [14] but variably detectable in adult cohorts before declining in association with post-burn immunosuppression [36].

A TNF-α peak occurred within the first 2–3 days post-burn, with smaller magnitudes than IL-6 or IL-8 [6,16]. Both TNF-α and IL-1β were markedly higher in burn wound exudates than in plasma, underscoring the wound as a persistent cytokine reservoir sustaining systemic inflammation [17,34].

IL-2 and IL-7 showed variable patterns; network analysis revealed strong correlations among IFN-γ, IL-2, IL-4, IL-7, IL-12p70, and IL-17, consistent with coordinated T-helper responses [20]. In mechanically ventilated adults with burns, IL-6 and IL-8—but not IL-7—were associated with mortality and VAP [21]. IL-13 showed no consistent discriminatory value between survivors and non-survivors in large pediatric cohorts [14]. It rose acutely but declined thereafter, mirroring the transition toward persistent inflammation-immunosuppression (PIICS) [36].

Network analyses demonstrated clustering of IL-12p70 with IFN-γ, IL-2, IL-4, IL-7, and IL-17, suggesting an integrated immune response rather than isolated mediator effects [20].

### 3.2. Anti-Inflammatory and Counter-Regulatory Cytokines

IL-10 peaked on day 1 post-burn, then declined. Higher levels correlated with TBSA and sepsis [6,14]. Counter-regulatory mediators like IL-10, IL-4, and G-CSF attempt to restore balance. However, age and sex modulate their efficacy: children often overshoot into hyperinflammation, while elderly patients mount a blunted anti-inflammatory response, both predisposing to adverse outcomes [14,37,38]. Markedly elevated G-CSF was found in severely burned children, especially non-survivors [14,16]. By contrast, in elderly patients, G-CSF responses were blunted, together with IL-6 and MCP-1, a pattern associated with impaired neutrophil mobilization and increased infection risk [22,38].

Regarding IFN-γ, early increases were observed after major burns, with prognostic differences reported in pediatric cohorts [16]. In addition, other pediatric studies confirmed outcome-related cytokine alterations, including elevated TNF-α and IL-6 and reduced leptin and growth factors [13]. In children, IFN-γ-dominated T helper 1 (Th1) responses persisted over weeks, reflecting heightened but maladaptive immune activation [31]. IL-4 and IL-10, produced in the context of Th2 responses and mast cell activation, contribute to the anti-inflammatory balance after burns. Their modulation has been linked to sex-specific differences, with hormonal influences such as estrogens suggested as possible contributors [37]. Both IL-4 and IL-17 cytokines showed age- and time-dependent variations. During the first week, adults had higher IL-4 and IL-17 than children [16]. Later changes were inconsistent, without a clear trajectory [19]. In pediatric cohorts, IL-4 and IL-17 were elevated at certain time points in non-survivors compared with survivors, but no consistent discriminatory pattern emerged [39]. IL-17 secretion by Th17 and γδ T-cells increases rapidly after burn injury, facilitating early neutrophil recruitment and pathogen defense. However, prolonged IL-17/Th17 responses have been associated with immune dysfunction and risk of sepsis when unregulated [40].

Persistent Th1/Th17 skewing illustrates how early host defense can shift into maladaptation: IFN-γ and IL-17 responses remain dominant in pediatric cohorts, reflecting immune overdrive that paradoxically coincides with functional exhaustion [31,40].

### 3.3. Balance of Pro- and Anti-Inflammatory Factors

Temporal profiling showed that both pro-inflammatory cytokines (IL-6, IL-8, IL-1β, MCP-1, TNF-α) and anti-inflammatory mediators (IL-10, IFN-γ, IL-4, IL-17) increase markedly during the first week post-burn, followed by a trend toward normalization over ~5 weeks, with age-dependent kinetics [16]. In survivor vs. non-survivor trajectories, non-survivors exhibited persistent elevations of IL-6, IL-8, G-CSF, and MCP-1 (up to 10,000-fold differences) associated with sepsis and multi-organ failure [19]. Network analysis revealed strong interconnections among various T-helper cytokines (IFN-γ, IL-2, IL-4, IL-7, IL-12p70, and IL-17), indicating that post-burn immune responses are driven by coordinated cytokine networks rather than by isolated mediators [20]. In patients with severe burns, persistent systemic inflammation was accompanied by a sustained release of immature neutrophils and shifts in T cell subsets toward a more pro-inflammatory phenotype, highlighting disturbances in the balance of immune regulation [29]. Together with evidence of complement activation and neutrophil extracellular trap formation, cytokine measurements from burn wound exudates and tissue secretome profiling indicate that systemic inflammatory profiles are tightly linked to their local source in the wound bed [17,34]. Cytokine levels in wound exudates (IL-1β, IL-8, TNF-α, MCP-1, GM-CSF) exceeded matched plasma, confirming the wound as a persistent inflammatory source [17].

### 3.4. Hematologic Ratios and Systemic Inflammation Indices

NLR is strongly associated with mortality in burn patients [8,27]. In the Vietnamese cohort analyzed by Hung et al. [27], a day-7 neutrophil-to-lymphocyte ratio (NLR) ≥ 14.13 was identified as an independent prognostic cut-off for mortality (sensitivity 75%, specificity 83%), remaining significant after adjustment for age, burn size, and inhalation injury. Additional hematologic-derived indices measured at admission also showed prognostic value: LPR on day 1 = 6.37 (AUC 0.695); NLPR on day 3 = 8.06 (AUC 0.794); and NLPR on day 7 = 3.84 (AUC 0.814). When these indices were combined with the Prognostic Burn Index, the accuracy of prognosis improved markedly reaching an AUC 0.994 [28].

### 3.5. Acute-Phase Reactants and Other Mediators

A meta-analysis identified a CRP threshold around 71 mg/L associated with adverse outcomes in burn cohorts [41]. In our included studies, Tang et al. reported that PCT > 2.8 ng/mL was associated with higher mortality and infectious complications, although PCT was not an independent predictor once adjusted with RDW, age and TBSA [24]. These data support the potential of acute-phase reactants as risk stratifiers, while underscoring the need for validation across assay platforms and timepoints.

### 3.6. Complement Activation and Neutrophil-Related Mediators

C3a was elevated early after injury but did not consistently discriminate severity [9]. Plasmin-driven pathways bridge coagulation and inflammation, fueling burn-induced Systemic Inflammatory Response Syndrome (SIRS) alongside complement activation [9,10]. Among neutrophil extracellular trap markers, CitH3 and neutrophil elastase—but not MPO or C3a—tracked with higher Abbreviated Burn Severity Index (ABSI), underscoring complement-neutrophil interplay [9]. Neutrophils in impending sepsis display spontaneous migration 24–48 h before clinical diagnosis suggesting they could be a potential early infection biomarker [11]. Beyond C3a, activation fragments such as C5a and C4d have been implicated in neutrophil chemotaxis and endothelial injury, underscoring that multiple complement pathways are engaged in parallel during the acute burn response [42]. Humoral-cellular cross-talk closes the loop: complement activation (C3a) and neutrophil extracellular traps (CitH3, NE) connect coagulation, innate immunity, and tissue injury, marking the transition from acute SIRS to chronic PIICS, persistent inflammation, immunosuppression and catabolism syndrome [9,10,11].

## 4. Discussion

This systematic review synthesized data from 24 eligible studies evaluating cytokines, hematologic indices, and complement activation in burn patients. Overall, the findings highlight that the post-burn immune response represents a dynamic balance between pro- and anti-inflammatory forces, with magnitude and persistence—rather than single time points—shaping clinical outcomes. Recent evidence emphasizes that burn injury induces not only a systemic cytokine storm but also persistent local inflammation in skin and adipose tissue, demonstrated in mechanistic animal models [33] and confirmed in skin biopsies [34].

Across multiple cohorts, pro-inflammatory cytokines (IL-6, IL-8, IL-1β, MCP-1, TNF-α) rise within 24–72 h and track with burn size/severity; anti-inflammatory mediators (IL-10, G-CSF, IFN-γ, IL-4, IL-17) also increase early but follow less uniform, often transient kinetics [14,16,43]. Notably, in adult burn patients, classic IL-6 signaling—rather than trans-signaling—was directly associated with worse survival, underscoring IL-6 as more than a generic marker but a potential pathogenic driver [23].

Survivors vs. non-survivors diverge primarily by how long inflammation remains unchecked: non-survivors show sustained elevations of IL-6, IL-8, G-CSF and MCP-1 well beyond the acute phase—sometimes several orders of magnitude higher—while in survivors, those gradually normalize [14,19]. Nevertheless, several studies also reported that higher early cytokine concentrations, particularly IL-6 and IL-8 within the first 24–72 h post-burn, were associated with increased mortality and infectious complications [14,16,23]. Therefore, both the initial magnitude and the sustained persistence of the inflammatory response appear to influence outcomes, highlighting the need to consider the entire temporal trajectory rather than isolated time points. Cytokine imbalance also stratifies pulmonary risk. In intubated burn patients, IL-8 (but not IL-6 after adjustment) independently predicted ventilator-associated pneumonia (VAP) and mortality; IL-8-centric risk persisted at serial time points [21].

Burn injury exhibits a biphasic pattern: an initial overdrive followed by immune suppression, fostering infection. Persistent systemic inflammation has been linked to influx of immature neutrophils and shifts in T-cell subsets—reduced adaptive competence, whereas mechanistic studies in animal models suggest that dampening early overactivation improves survival [29,44]. Counter-regulation is evident through increases in sTNFR-I/II and IL-1 receptor antagonist (IL-1Ra) alongside IL-10/IL-13 after burn—biomarkers of the host’s attempt to dampen the cascade and potential targets for selective immunomodulation [15].

Early protein C activation mirrored injury severity and higher inflammatory burden, associating with worse outcomes—reinforcing the link between coagulation pathways and systemic inflammation [30]. Immunopathology extends to complement and neutrophil activity [9]. Anaphylatoxins released during complement activation (C3a, C5a) are elevated after burn injury, but their prognostic discrimination has been variable across studies [9,42]. In adults, C3a levels remained elevated for up to four weeks, but did not consistently discriminate burn severity. By contrast, NET-osis markers such as citrullinated histone H3 (CitH3) and neutrophil elastase were significantly higher in patients with ABSI > 9, whereas MPO and C3a failed to separate severity strata [9], supporting the contribution of neutrophil activation to outcome severity.

Beyond C3a, downstream complement fragments such as C5a and C4d have been implicated in neutrophil chemotaxis and endothelial injury, pointing to simultaneous activation of multiple complement pathways during acute burn response [9,42]. Bedside markers offer practical prognostic leverage. NLR was strongly associated with mortality across studies [8], and in a ≥20% TBSA cohort, day-7 NLR = 14.13 independently discriminated mortality (Se 75%, Sp 83%) [27]. Composite systemic indices—SII, NLPR, LPR—showed independent values and, combined with the Prognostic Burn Index, reached AUC approximately 0.994, suggesting that simple hematology can power high-accuracy risk tools [28].

Meta-analyses have reported clinically meaningful cut-offs such as PCT approximately 1.77 ng/mL and CRP approximately 71 mg/L [41]. In contrast, a single-center study observed that PCT values above approximately 2.8 ng/mL were associated with adverse outcomes [24], indicating variability across cohorts but supporting the prognostic value of these markers for early triage. These thresholds were reported as data-driven cut-offs in the respective studies and were not predefined in our protocol. The observed variability in PCT levels (1.77 vs. 2.8 ng/mL) likely reflects differences in study design, patient populations, and analytical assays, yet all studies consistently associated higher PCT levels with adverse outcomes. Additional single-center data support CRP elevation distinguishing infectious vs. non-infectious inflammation in the burn context [13,41].

With regard to age-dependent biology, children often mount stronger and more sustained cytokine responses (weeks), while elderly patients demonstrate blunted acute-phase dynamics (IL-6, MCP-1, G-CSF), consistent with immunosenescence [22,38,39], which may partly explain their increased susceptibility to infection and delayed recovery.

Cytokines rarely act alone. Correlation/network analyses demonstrate clustering of T-helper cytokines (IFN-γ, IL-2, IL-4, IL-7, IL-12p70, IL-17)—evidence that adaptive polarization unfolds as a coordinated program, not independent fluctuations [20,43]. At the tissue level, burn-injured skin shows a prolonged local acute inflammatory response, bridging local and systemic axes [34]. Emerging frameworks propose multi-marker signatures that integrate cytokines, chemokines, complement and cell-derived mediators for earlier diagnosis of burn-induced pathologies [45].

Inflammatory responses in severe burns can be viewed as a dynamic continuum beginning with a systemic inflammatory response syndrome (SIRS) phase, followed by a compensatory anti-inflammatory response (CARS), and, in some patients, progressing to a state of persistent inflammation, immunosuppression, and catabolism (PIICS). These overlapping stages represent an ongoing imbalance between sustained innate activation and insufficient adaptive recovery, which helps explain the long-term immune vulnerability observed after the acute phase of burn injury. Understanding this trajectory may support the development of immune-phase-specific therapeutic strategies aimed at restoring homeostasis [36]. Advanced clustering analyses of biomarker panels confirm that composite immune profiles—combining cytokines, systemic indices, and complement/NET markers—outperform single thresholds in predicting mortality [45]. It is important to note that while experimental animal studies have provided valuable mechanistic insights (e.g., cytokine kinetics, inflammasome activation), all clinical conclusions in this review are derived from human studies.

Persistent hyperinflammation, secondary immune dysfunction that promotes infections, and age-specific vulnerabilities each contribute to mortality [22,36]. Simple markers such as NLR, SII, NLPR, LPR, PCT, and CRP are useful for bedside risk assessment [27,28], whereas more detailed analyses of cytokines, complement factors, and NETs can refine our understanding of the underlying immune mechanisms [9]. Future work should focus on composite immune signatures that quantify both magnitude and direction of inflammatory change over time, enabling earlier, personalized immunomodulation [45]. Recent updates reinforce this direction: systematic reviews of sepsis biomarkers highlight the need for multi-marker panels to improve early diagnosis [46], while mechanistic studies confirm NF-κB as a central regulator of burn-induced inflammation [47]. Together, these findings underscore the rationale for early, targeted immunomodulation.

From a practical perspective, the identification of critical thresholds for markers such as NLR, PCT, CRP, and systemic inflammatory indices highlights the value of integrating easily accessible laboratory tests with more advanced cytokine profiling. This dual approach offers the possibility of stratifying patients not only by the extent of burn injury but also by their individual immunological trajectory. The incorporation of such markers into clinical scoring systems may significantly improve early prognostication and guide decision-making in intensive care. Future clinical practice may benefit from integrating immune markers into existing scoring systems, for example ABSI, thereby bridging basic immunology with pragmatic bedside risk stratification. In summary, integrating immune biomarkers into existing clinical scoring systems could help shift burn care from reactive to proactive, enabling early identification of high-risk patients and guiding personalized immunomodulation.

Beyond diagnostic and prognostic applications, future research should explore immunomodulatory strategies tailored to the evolving immune phases after burn injury. Early-phase interventions could aim to attenuate excessive cytokine activation and limit tissue injury, whereas later strategies may support immune recovery and prevent prolonged immunosuppression. Longitudinal immune monitoring and multicenter validation of biomarker-based risk models will be crucial to identify optimal intervention windows. Such phase-specific approaches could bridge mechanistic insight and clinical practice, ultimately improving survival and long-term outcomes in severe burns.

### Limitations

This systematic review has several limitations. First, the included studies showed considerable heterogeneity in terms of patient populations (pediatric vs. adult), burn severity, timing of sample collection, and laboratory methods used for cytokine and biomarker measurement. Furthermore, variability in analytical platforms (e.g., ELISA, multiplex bead assays, and chemiluminescent immunoassays) and detection limits across studies likely contributed to inter-study differences in reported cytokine concentrations, affecting comparability and quantitative precision. A further contributor to this heterogeneity is the lack of a standardized definition of “severe burns,” with TBSA thresholds ranging between >20% and >40% across studies. While we accepted the authors’ definitions to avoid unnecessary exclusion of relevant data, this variability may have introduced bias and limits direct cross-study comparisons. This heterogeneity precluded a formal meta-analysis and limited the ability to establish precise quantitative thresholds across cohorts.

Despite this, we performed a structured narrative synthesis to identify consistent patterns across studies evaluating similar markers (e.g., IL-6, CRP, NLR). This approach allowed us to qualitatively assess convergence of findings despite methodological and population differences. The main sources of heterogeneity included variations in study design, timing of sampling, and definitions of clinical outcomes.

Second, most available studies were single-center and had relatively small sample sizes, which may reduce generalizability.

Third, several biomarkers were assessed at different time points, making temporal comparisons difficult. Importantly, there is still a scarcity of data on the dynamics of cytokine and biomarker changes over time after burn injury, as well as on the interplay between pro- and anti-inflammatory mediators depending on the day of burn. These gaps suggest that the temporal trajectories and interactions of immune markers are not yet fully described in the literature.

Additionally, most included studies provided limited information on patient comorbidities (such as diabetes, cardiovascular disease, or obesity) and treatment variables (e.g., fluid resuscitation, antibiotics, or immunomodulatory therapy), which are known to influence inflammatory responses and outcomes. The absence of consistent adjustment for these factors represents another potential source of bias and limits the ability to isolate the independent prognostic value of specific biomarkers.

Finally, publication bias cannot be excluded, as studies with negative or inconclusive results may have remained unpublished. Because no formal publication bias assessment (e.g., funnel plot or Egger’s test) could be performed due to the absence of pooled quantitative data, the potential overrepresentation of positive findings must be considered when interpreting the overall prognostic consistency. Despite these limitations, the review provides a comprehensive synthesis of current evidence and highlights consistent patterns that are clinically and biologically relevant. Future systematic studies with standardized protocols and harmonized cytokine assays are warranted to enable meta-analysis and validate prognostic thresholds across burn populations.

## 5. Conclusions

This systematic review demonstrates that the inflammatory response to burn injury is not a simple sequence of events but rather a dynamic and unstable equilibrium between pro- and anti-inflammatory forces. The evidence indicates that clinical outcomes are influenced by both the initial magnitude of cytokine release—reflecting the early systemic insult—and the persistence of dysregulated inflammation or failure of compensatory mechanisms. Survivors tend to normalize their cytokine and biomarker profiles, whereas in non-survivors these abnormalities persist as sustained hyperinflammation or paradoxical immune suppression, ultimately preceding death. This immune dysregulation follows a trajectory rather than a single peak event—an initial surge, followed by either resolution and recovery or sustained dysfunction leading to sepsis and organ failure.Mechanistic findings involving complement activation, neutrophil extracellular trap formation, and soluble receptor pathways reinforce the notion that burn injury represents a systemic immune disorder engaging both innate and adaptive networks. Emerging evidence—from NF-κB-driven inflammation to complement and NET activation—supports the view that composite biomarker panels, rather than isolated thresholds, will guide the next generation of prognostic and therapeutic strategies. Future research should aim to move beyond single-biomarker approaches and develop composite biomarker panels that capture the multidimensional nature of this response. Such panels, once validated, could transform burn care by enabling early risk stratification and guiding personalized immunomodulatory strategies to improve survival and recovery in patients with severe burns.

## Figures and Tables

**Table 1:** Cytokine studies.

Author/Year	Study Characteristics	Key Biomarkers (Measured)	Main Findings
Finnerty et al., 2006 [6]	Severely burned pediatric patients (n = 19; >40% TBSA, no sepsis or inhalation injury) vs. healthy controls (n = 14); serial sampling up to 5 weeks	17 cytokines (IL-1α, IL-2, IL-4, IL-5, IL-6, IL-7, IL-8, IL-10, IL-12p70, IL-13, IL-17, IFN-γ, MCP-1, MIP-1α, G-CSF, GM-CSF, TNF-α)	Fifteen out of 17 cytokines were significantly higher during week 1; GM-CSF peaked in week 2; TNF-α showed no significant difference vs. controls. Levels trended down but remained above controls by week 5; authors suggested a therapeutic window in the first week.
Abdel-Hafez et al., 2007 [13]	Children with burns (n = 42; sepsis 47.6%; mortality 28.6%) vs. controls (n = 26); baseline and day 8	TNF-α, IL-6, PCT, CRP, leptin, bFGF, TGF-α	Burn patients had higher TNF-α, IL-6, PCT, CRP, leptin vs. controls. In sepsis and in non-survivors, IL-6, TNF-α, PCT and CRP were further increased, while leptin, bFGF and TGF-α were lower. IL-6 and PCT had prognostic value for sepsis and mortality.
Finnerty et al., 2007 [14]	Pediatric burn patients (n = 44; septic fatal n = 15 vs. non-septic survivors n = 29); admission samples	7 cytokines (IL-6, IL-8, IL-10, IFN-γ, TNF-α, IL-12p70, GM-CSF)	All seven cytokines were significantly higher in septic fatal patients at admission. In multivariable modeling, the best predictor of fatal sepsis combined elevated IL-6 and IL-12p70 with relatively decreased TNF-α (AUC 0.92); the TNF-α decrease reflects the model coefficient although raw TNF-α was higher in fatal cases.
Sikora et al., 2008 [15]	Burned children (n = 17) vs. controls (n = 20); early phase (6–24 h) and post-therapy	sTNFR-I, sTNFR-II, IL-1Ra, IL-10, IL-13, neutrophil ROS	Burn patients showed increased IL-10, sTNFR-I, sTNFR-II, IL-1Ra, and a non-significant trend toward higher IL-13, with reduced neutrophil ROS. After therapy, inhibitors and IL-10/IL-13 decreased while ROS increased. Poor outcome (hypovolemic shock) was associated with persistently high inhibitors/IL-10/IL-13 and low ROS.
Finnerty et al., 2008 [16]	Adults (n = 25) vs. children (n = 24), all survivors of flame burns (>20% TBSA in adults, >40% in children)	22 cytokines (IL-1α, IL-1β, IL-2, IL-4, IL-5, IL-6, IL-7, IL-8, IL-10, IL-12, IL-13, IL-15, IL-17, IL-18, IFN-γ, TNF, MCP-1, MIP-1α, IP-10, GM-CSF, G-CSF, eotaxin)	During the first week, adults had significantly higher IFN-γ, IL-10, IL-17, IL-4, IL-6 and IL-8. Children showed later increases in IL-1β (week 2), IL-18 (week 3) and IL-1α (days 21–66). GM-CSF was consistently lower in children at all time points. Additional differences were observed for eotaxin, G-CSF, IL-13, IL-15, IP-10, MCP-1 and MIP-1α, whereas IL-12, IL-2, IL-7 and TNF did not differ between groups.
Mikhal’chik et al., 2009 [17]	Children with severe burns (n = 19); paired plasma vs. wound exudate	IL-1β, IL-1Ra, IL-2, IL-6, IL-8, IL-10, TNF-α, IFN-γ, MCP-1, MIP-1α, G-CSF, GM-CSF, PDGF-BB, VEGF	IL-1β, IL-1Ra, IL-8, MCP-1, MIP-1α, TNF-α, GM-CSF higher in exudate; IL-2, IL-6, IL-10, G-CSF, IFN-γ, PDGF-BB, VEGF higher in plasma. No plasma-exudate correlation; burn wounds are a local source of pro-inflammatory cytokines, with MPO correlating to MIP-1α, TNF-α, GM-CSF.
Csontos et al., 2010 [18]	Adults with severe burns (≥20% TBSA), n = 39; serial plasma measurements on days 1–6; comparison survivors (n = 21) vs. non-survivors (n = 18)	IL-1β, IL-6, IL-8, IL-10, TNF-α, IL-12p70 (undetectable); both unstimulated and PMA-stimulated plasma	IL-10 consistently higher in non-survivors and strongest predictor of ICU mortality (admission ≥ 14 pg/mL: sensitivity ~85%, specificity ~84%). IL-6 and IL-8 rose later and were higher in non-survivors. Pro/anti-inflammatory ratios (e.g., IL-6/IL-10) had limited prognostic value compared with IL-10 alone; early excess of IL-10 indicated poor outcome.
Jeschke et al., 2014 [19]	Pediatric burn cohort (n = 230; >30% TBSA); survivors vs. non-survivors; trajectories followed up to 180 days	IL-6, IL-8, G-CSF, MCP-1 (within a broader 17-plex panel), plus CRP, glucose, insulin, BUN, creatinine, bilirubin, and metabolic/REE measures	Non-survivors had persistently higher IL-6, G-CSF, MCP-1 (from early on) and IL-8 (diverging after day 8–10), along with higher CRP, metabolic and organ dysfunction markers, and more pronounced hypermetabolism. Distinct inflammatory/metabolic trajectories separated survivors from non-survivors.
Hur et al., 2015 [20]	Adults with major burns (n = 67; ≥20% TBSA); serum days 1 and 3; survivors vs. non-survivors	27-plex cytokines (incl. IL-1Ra, IL-6, IL-8, IL-10, MCP-1, PDGF-BB, VEGF)	Non-survivors had higher IL-1Ra, IL-6, and MCP-1 on day 1, while by day 3 only PDGF-BB was lower. Compared with controls, several cytokines were elevated overall, with early IL-1Ra, IL-6, and MCP-1 emerging as the strongest indicators of poor outcome.
Shelhamer et al., 2015 [21]	Adults with severe burns requiring mechanical ventilation (n = 62; with/without inhalation injury); plasma on days 0, 3, 7	IL-1α, IL-6, IL-8, GM-CSF, TNF-α	IL-6 and IL-8 were higher in non-survivors at day 7. In multivariable logistic regression, IL-8 independently predicted the composite of death or VAP with odds ratios of 7.9 (day 0), 26 (day 3) and 7.3 (day 7); IL-6 lost significance after adjustment. The majority of IL-1α, GM-CSF and TNF-α values were below the limit of quantification.
Jeschke et al., 2015 [22]	Adults (<65 years, n = 94) vs. elderly (≥65 years, n = 36) with severe burns; single-center cohort, subset of a larger registry (2796 total; 1461 analyzed 2006–2015)	Cytokines/chemokines (IL-1β, IL-6, IL-8, IL-10, TNF-α, MCP-1, GM-CSF, etc.), metabolic markers (REE, glucose, insulin, c-peptide), and wound-healing mediators	Elderly patients exhibited an early blunted but later sustained inflammatory response, delayed yet persistent hypermetabolism, impaired insulin secretion, and impaired repair processes, correlating with higher organ failure and mortality despite similar infection rates compared with younger adults.
Matsuura et al., 2019 [7]	Adults with major burns (n = 38; ≥20% TBSA); serial serum samples days 1–30; survivors vs. non-survivors	11 cytokines: IFN-α, IFN-γ, IL-1β, IL-6, IL-8, IL-10, IL-12/IL-23p40, IL-17A, TNF-α, MCP-1, IL-4	IL-6, IL-8, IL-10, and MCP-1 were elevated early and formed a prognostic cytokine network. Higher levels in non-survivors correlated with SOFA and 28-day mortality; ROC analyses showed IL-6+IL-10 provided the best predictive accuracy.
Rehou et al., 2023 [23]	Adults with severe burns (n = 86), admitted within 7 days post-injury; grouped by IL-6/sIL-6R ratio (<0.185 vs. ≥0.185)	IL-6, soluble IL-6R; additional panel: IL-1β, IL-8, IL-17, TNF-α, IL-1Ra, IL-10, G-CSF, GM-CSF, MCP-1, MIP-1α/β, IL-2	High-ratio patients (≥0.185, n = 46) had larger burns, more inhalation injury, higher IL-6 (but not sIL-6R), and elevated IL-1β, IL-8, TNF-α, IL-10, G-CSF, MCP-1, MIP-1β, IL-2. They showed higher mortality (26% vs. 3%), more acute kidney injury and graft loss, and longer hospitalization, indicating that predominant classic IL-6 signaling is associated with poor outcomes.

TBSA—Total Body Surface Area, IL—Interleukin, TNF-α—Tumor Necrosis Factor alpha, IFN-γ—Interferon gamma, IFN-α—Interferon alpha, MCP-1—Monocyte Chemoattractant Protein-1, MIP-1α—Macrophage Inflammatory Protein-1 alpha, MIP-1β—Macrophage Inflammatory Protein-1 beta, IP-10—Interferon gamma-induced protein 10 (CXCL10), G-CSF—Granulocyte Colony-Stimulating Factor, GM-CSF—Granulocyte-Macrophage Colony-Stimulating Factor, CRP—C-reactive protein, PCT—Procalcitonin, bFGF—basic Fibroblast Growth Factor, TGF-α—Transforming Growth Factor alpha, IL-1Ra—Interleukin-1 receptor antagonist, sTNFR-I—soluble TNF receptor I, sTNFR-II—soluble TNF receptor II, ROS—Reactive Oxygen Species, PDGF-BB—Platelet-Derived Growth Factor-BB, VEGF—Vascular Endothelial Growth Factor, BUN—Blood Urea Nitrogen, REE—Resting Energy Expenditure, AUC—Area Under the Curve, SOFA—Sequential Organ Failure Assessment score, VAP—Ventilator-Associated Pneumonia, sIL-6R—soluble Interleukin-6 Receptor, ICU—Intensive Care Unit.

**Table 2:** Hematological ratios and acute-phase reactants studies.

Author/Year	Study Characteristics	Key Biomarkers (Measured)	Main Findings
Tang et al., 2023 [24]	Adults with severe burns in China (n = 148; TBSA ≥ 30%; admission samples; 2017–2022)	PCT, RDW	Both higher in non-survivors. RDW (with age and TBSA) was an independent predictor; PCT was not. Higher values associated with increased 90-day mortality.
Juárez and Sánchez, 2023 [25]	Observational, retrospective, Mexico (CENIAQ); n = 85 (2020–2022); severe burns; NLR at admission; outcome: mortality	NLR	Admission NLR was higher in non-survivors. Suggested as a mortality predictor, though no ROC cut-off or multivariable analysis was provided.
Zaldívar Castillo and Palacios, 2024 [26]	Prospective, Cuba; 2022; n = 36 “Grandes Quemados”, 19–60 years; samples at 72h and day 6; outcomes at discharge	NLR (INL)	NLR rose by 72h and remained high at day 6. Higher values in poor prognosis and non-survivors. Directly related to burn severity and survival.
Hung et al., 2024 [27]	Retrospective, Vietnam; n = 245 adults (TBSA ≥ 20%), admitted <24h; NLR at admission, day 3, day 7; excluded deaths < 7 days	NLR	NLR elevated at all times; significantly higher in non-survivors. Only day 7 NLR independently predicted mortality, alongside age, burn size and inhalation injury.
Li et al., 2024 (Burns) [28]	Retrospective, China; n = 135 extensive burns; CBC indices at days 1/3/7; in-hospital mortality	LPR, NLR, NMR, MLR, NLPR, SII, SIRI	Certain derived indices (LPR day 1, NLPR days 3 and 7) independently predicted mortality. Higher values linked to worse survival; combining indices with burn severity improved prognostic performance.
Awad et al., 2024 (meta-analysis) [8]	Meta-analysis of admission NLR for mortality prediction; 9 studies, n = 1837; heterogeneity and meta-regression performed	NLR (admission)	Across studies, admission NLR consistently higher in non-survivors. Despite heterogeneity, supports NLR as simple and useful marker for early mortality risk stratification in burns.

TBSA—Total Body Surface Area, PCT—Procalcitonin, RDW—Red Cell Distribution Width, NLR—Neutrophil-to-Lymphocyte Ratio, ROC—Receiver Operating Characteristic, INL—Índice Neutrófilo-Linfocito (Spanish for NLR), CBC—Complete Blood Count, LPR—Lymphocyte-to-Platelet Ratio, NMR—Neutrophil-to-Monocyte Ratio, MLR—Monocyte-to-Lymphocyte Ratio, NLPR—Neutrophil-to-Lymphocyte-to-Platelet Ratio, SII—Systemic Immune-Inflammation Index, SIRI—Systemic Inflammation Response Index.

**Table 3:** Immune cell function studies.

Author/Year	Study Characteristics	Key Immune Markers/Assays (Measured)	Main Findings
Jones et al., 2014 [11]	Adults with major burns; prospective longitudinal sampling around sepsis onset (n = 13; 71 samples)	Neutrophil spontaneous migration (functional assay)	Aberrant spontaneous migration specific for sepsis, detectable 1–2 days (and significantly ≥3 days) before clinical diagnosis; values decreased after antibiotics. Averaging two consecutive samples from the same patient improved discrimination (AUC ~0.80).
Mulder et al., 2021 [29]	Adults with severe burns; n = 20 ICU patients (TBSA ≥ 15%) vs. n = 20 healthy controls; longitudinal PBD 0–39	Flow cytometry: neutrophil maturation, monocyte subsets, CD4+ T-cell subsets; plasma cytokines	Burn patients showed persistent elevation of IL-6, IL-8, MCP-1, MIP-1β, MIP-3α, a sustained influx of immature neutrophils, expansion of monocyte subsets, and by week 2, increased CCR4+/CCR6+ CD4 T cells and Tregs, reflecting a prolonged pro-inflammatory state.
Laggner et al., 2022 [9]	Adults with thermal burns; n = 32 (TBSA > 10%) vs. 8 controls; samples daily (week 1) then weekly to day 28; severity stratified by ABSI, burn depth, SOFA, APACHE II	CitH3, neutrophil elastase (NE), MPO, C3a	CitH3 and NE higher in severe burns (ABSI ≥ 9, 3rd-degree); MPO/C3a elevated vs. controls but less discriminatory. NE also linked to higher SOFA, MPO/CitH3 to higher APACHE II. Markers did not predict mortality.
Zhao et al., 2022 [30]	Prospective, adults with severe burns; n = 86; serial sampling from admission to day 21	APC (APC-PCI), PC, APC/PC (PC activation), sEPCR; CRP, IL-6, IL-8	Early protein C activation reflected burn severity, was linked to stronger inflammation, and associated with worse clinical outcomes.
Langley et al., 2024 [31]	Pediatric burn patients; n = 6 vs. n = 4 controls; longitudinal immune profiling up to 18 months post-burn	T-cell phenotypes; macrophage activity markers	Pro-inflammatory T-cell profiles remained elevated up to 18 months, and macrophage activity was enhanced early after burn with some persistence later, indicating prolonged immune activation.

AUC—Area Under the Curve, ICU—Intensive Care Unit, TBSA—Total Body Surface Area, PBD—Post-Burn Day, IL—Interleukin, MCP-1—Monocyte Chemoattractant Protein-1, MIP-1β—Macrophage Inflammatory Protein-1 beta, MIP-3α—Macrophage Inflammatory Protein-3 alpha, CCR4—C-C chemokine receptor type 4, CCR6—C-C chemokine receptor type 6, Tregs—Regulatory T cells, ABSI—Abbreviated Burn Severity Index, SOFA—Sequential Organ Failure Assessment, APACHE II—Acute Physiology and Chronic Health Evaluation II, CitH3—Citrullinated Histone H3, NE—Neutrophil Elastase, MPO—Myeloperoxidase, C3a—Complement component 3a, APC—Activated Protein C, APC-PCI—Activated Protein C-Protein C Inhibitor complex, PC—Protein C, sEPCR—soluble Endothelial Protein C Receptor, CRP—C-reactive protein.

## Data Availability

No new data were created or analyzed in this study. Data sharing is not applicable to this article.

## Supplementary Materials

The following supporting information can be downloaded at: https://www.mdpi.com/article/10.3390/ijms262010131/s1.

## Abbreviations

The following abbreviations are used in this manuscript:

ABSI	Abbreviated Burn Severity Index
APACHE II	Acute Physiology and Chronic Health Evaluation II
APC	Activated Protein C
APC-PCI	Activated Protein C-Protein C Inhibitor complex
AUC	Area Under the Curve
BUN	Blood Urea Nitrogen
bFGF	basic Fibroblast Growth Factor
C3a	Complement component 3a
CARS	Compensatory Anti-inflammatory Response Syndrome
CBC	Complete Blood Count
CCL2 (MCP-1)	Monocyte Chemoattractant Protein-1
CCL4 (MIP-1β)	Macrophage Inflammatory Protein-1 beta
CCR4	C-C chemokine receptor type 4
CCR6	C-C chemokine receptor type 6
CitH3	Citrullinated Histone H3
CRP	C-reactive Protein
G-CSF	Granulocyte Colony-Stimulating Factor
GM-CSF	Granulocyte-Macrophage Colony-Stimulating Factor
ICU	Intensive Care Unit
IFN-α	Interferon alpha
IFN-γ	Interferon gamma
IL	Interleukin
IL-1Ra	Interleukin-1 receptor antagonist
INL	Índice Neutrófilo-Linfocito (Spanish for NLR)
LPR	Lymphocyte-to-Platelet Ratio
MIP-1α	Macrophage Inflammatory Protein-1 alpha
MIP-3α	Macrophage Inflammatory Protein-3 alpha
MLR	Monocyte-to-Lymphocyte Ratio
MPO	Myeloperoxidase
NE	Neutrophil Elastase
NET	Neutrophil Extracellular Trap
NLR	Neutrophil-to-Lymphocyte Ratio
NLPR	Neutrophil-to-Lymphocyte-to-Platelet Ratio
NMR	Neutrophil-to-Monocyte Ratio
PBD	Post-Burn Day
PC	Protein C
PCT	Procalcitonin
PDGF-BB	Platelet-Derived Growth Factor subunit B
PIICS	Persistent Inflammation, Immunosuppression and Catabolism Syndrome
PRISMA	Preferred Reporting Items for Systematic Reviews and Meta-Analyses
RDW	Red Cell Distribution Width
REE	Resting Energy Expenditure
ROC	Receiver Operating Characteristic
sEPCR	soluble Endothelial Protein C Receptor
SII	Systemic Immune-Inflammation Index
SIRS	Systemic Inflammatory Response Syndrome
sIL-6R	soluble Interleukin-6 Receptor
SIRI	Systemic Inflammation Response Index
SOFA	Sequential Organ Failure Assessment score
sTNFR	Soluble Tumor Necrosis Factor Receptor
TBSA	Total Body Surface Area
TGF-α	Transforming Growth Factor alpha
TNF-α	Tumor Necrosis Factor alpha
Tregs	Regulatory T cells
VAP	Ventilator-Associated Pneumonia
VEGF	Vascular Endothelial Growth Factor
